# From single course to comprehensive programme: Experiences developing and establishing an Office for Medical Education

**DOI:** 10.3205/zma001117

**Published:** 2017-10-16

**Authors:** Thomas Kollewe, Monika Sennekamp, Falk Ochsendorf

**Affiliations:** 1 J. W. Goethe-Universität Frankfurt, FB Medizin, Frankfurter Arbeitsstelle für Medizindidaktik, Frankfurt, Germany; 2 J. W. Goethe-Universität Frankfurt, FB Medizin, Dekanat, Frankfurt, Germany; 3 Universitätsklinikum Frankfurt, Klinik für Dermatologie, Venerologie und Allergologie, Frankfurt, Germany

**Keywords:** medical education, medical teaching

## Abstract

Since 2002 several individual courses in medical education have been offered by the Frankfurt University Medical School and its teaching hospital. This programme was expanded in 2011 into a comprehensive, structured programme covering the broad spectrum of topics surrounding teaching; the programme is housed within the Medical School as part of the Frankfurter Arbeitsstelle für Medizindidaktik (FAM). The following factors were critical to successful implementation: existing programmes in other German states (primarily Baden-Württemberg and North Rhine-Westphalia) with matching requirements, support from the Deans of Studies, anchoring attendance rules for medical education courses in the university rules and regulations governing who is qualified to lecture at the university level, and a sufficient number of faculty members interested in teaching as a discipline in itself. The programme’s core elements include a basic course for all newly hired faculty with teaching responsibilities and a modular design that allows individuals to focus on their particular interests and needs. Although the programme is largely mandatory, there is a high level of satisfaction and a lasting growth in knowledge among participants.

## Zusammenfassung

Am Fachbereich Medizin und dem Klinikum der Johann Wolfgang-Goethe-Universität Frankfurt existierten bereits seit 2002 mehrere einzelne medizindidaktische Kurse. Diese Aktivitäten wurden 2011 strukturiert, ein umfassendes Kursangebot, das das breite Spektrum an Themen rund um die Lehre abdeckt, wurde aufgebaut und unter dem Dach der Frankfurter Arbeitsstelle für Medizindidaktik (FAM) am Fachbereich institutionalisiert. Folgende Faktoren waren für die erfolgreiche Umsetzung ausschlaggebend: vorhandene Programme in anderen Bundesländern (v.a. Baden-Württemberg, Nordrhein-Westfalen) mit entsprechenden Vorgaben, die Unterstützung der Studiendekane, die Verankerung der Teilnahme an medizindidaktischen Kursen in der Habilitationsordnung sowie eine kritische Masse von an der Lehre interessierten Mitarbeiterinnen und Mitarbeitern. Kernelemente des Angebots sind ein Basiskurs für alle neu eingestellten wissenschaftlichen Angestellten mit Lehrverpflichtung und ein modularer Aufbau des Programms, der individuellen Präferenzen bzw. Erfordernissen entgegen kommt. Gleichwohl die Teilnahme am Kursprogramm überwiegend verpflichtend erfolgt, zeigt sich eine hohe Zufriedenheit und ein nachhaltiger Wissenszuwachs bei den Kursteilnehmerinnen und Kursteilnehmern.

## 1. Introduction

Across Germany the different teacher training programmes for teachers in medical education vary highly. In some cases, courses are offered as part of general higher education; both scope and content differ, as do the university-specific requirements concerning mandatory attendance (e.g. qualifications for university lecturers). While Baden-Württemberg and North Rhine-Westphalia have had certification programmes for many years, in other German states the development of similar programmes has been slower. Since the end of 2010 there has been an attempt via the Medical Teaching Network (MDN) to compare programmes as a strategy to encourage programme development in ways that aim for mutual recognition [1]. This has resulted in the previously isolated activities involving teacher training for university lecturers at Frankfurt University’s Medical School being transformed as of August 2011 into the first Hessian programme meeting MDN criteria and the establishment of the Frankfurter Arbeitsstelle für Medizindidaktik (FAM), an office for medical education.

## 2. Project description

### 2.1. Background and development

As early as 2002, a two-day education course (FiTT – Frankfurt interdisciplinary Tutor Training) was offered twice a year and required of all those seeking to lecture at the university level. In addition, there were other separate courses on specific topics, such as grading assessments or training sessions for lecturers teaching courses on clinical examination techniques.

Those involved in organising and teaching the courses felt that there was a fair amount of repetition and that potential benefits from synergies were lost due to a lack of coordination. With the goal of changing this and bundling activities into an overall concept, motivated faculty members at the Medical School and its teaching hospital founded a working group in 2008 to address the issue of teaching and education. Since members of this working group also served on the GMA committee dealing with human resource and organizational development and participated in meetings held by the newly established MDN, it was possible to follow and incorporate national developments. This working group presented its plans to the Academic Commission and was officially given the task of developing a programme.

The structure of FAM was developed during a conference retreat and is described in more detail in sections 2.2 through 2.5. External advice from Hille Lieverscheidt, who had worked to augment and expand a similar programme in North Rhine-Westphalia, proved helpful during this phase. The working group created a basic framework for institutionalization that encompassed the basic course and initial ideas for the advanced training courses. This concept was presented to the Academic Commission and the Faculty Council. The working group continued its course development when the Faculty Council decided to provide FAM the funding for one full-time employee and one part-time office staff member. The basic argument was that only a university staff member could convincingly teach the courses and oversee the programme. Other achievements included:

1. Requiring 120 credits to register as a university lecturer. The Medical School was persuaded by the fact that similar requirements existed in other German states, and that those who attained the lectureship qualification at the University of Frankfurt should not face disadvantages when applying for positions elsewhere in the country.
2. Requiring newly hired faculty members with teaching duties [2] to take the basic course (24 credits). The main arguments were that courses immediately prior to assuming teaching responsibilities were simply too late to apply theory to practice in meaningful ways, and that a good course can motivate participants to grapple more closely with teaching and education and to take advantage of other continuing education opportunities – even without any concrete intention to qualify as a university lecturer.

Without the unfailing and dedicated support of the then Dean of Studies, Prof. Frank Nürnberger, and the commitment of a sufficient number of other motivated people, none of this would have been possible. The rationale for and benefit of having such a programme was simply not understood by a large number of medical faculty members.

After permission to proceed was given by the Studies Committee, the major part of the advanced training courses was planned in detail within small groups. During another two-day-long conference retreat that included faculty members from the Department of Higher Education [https://ikh.uni-frankfurt.de/], the courses were critically revised in terms of content, teaching methods, and feasibility.

When planning and making decisions, both the situation in Frankfurt and the consensus paper issued at the time by the MDN on mutual programme recognition were taken into account [3]. This ensured not only recognition of courses taken in Frankfurt at other medical schools, but also contributed to quality assurance on the formal level. The first basic course was held at the end of 2010 and firmly established by FAM as people were hired to fill positions. The overall process had taken approximately two years.

#### 2.2. Course structure and design

The FAM programme in teaching medical education at Frankfurt University begins with a one-day-long basic course (24 credits), in which principles such as the importance and formulation of learning objectives and the psychology of learning are covered. The other courses in the programme are sequenced into three modules: “Course organization,” “Teaching and methodology,” and “Assessments.” There is also a fourth module that is an elective (see Table 1 (Tab. 1)).

Each of these advanced training courses consists of 24 credits (one credit being equal to 45 minutes), breaks down into one and a half days, and includes assignments before and after the course is held. An important aspect of teaching the advanced training courses is imparting methods and techniques that participants can best apply to their own teaching. For this reason the courses focus on outcome, meaning the focus goes beyond just reading introductory texts on pedagogy to centre on something useful which is reflected in both the pre- and post-course assignments. To this end, trainees are given the assignment of drafting introductory sequences for a lecture or multiple-choice questions for a seminar. During the courses, regular reference is made to these preparatory activities, and time is allotted for making revisions and improvements. The final practical application is ensured in the post-course assignment. For example, concrete ideas are generated for practical exams or lesson plans are written for a class session, then micro-taught and evaluated through peer feedback.

After completing the 120 credits (= basic course + one course each from modules II and III + 2 elective courses) participants receive a Certificate in Medical Education (*Zertifikat für Medizindidaktik*).

Instead of requiring overall certification (120 credits) right off for lecturers-to-be, in 2011 only the basic course was necessary. In 2012 the additional advanced training course was added, and in 2013 yet another. As of 2015 there are five courses that must be completed.

#### 2.3. Trainers

All courses are team-taught by two trainers with at least one of them having qualifications in (medical) education and the other being a medical doctor or natural scientist. In the FAM pool of trainers, the education experts have either a Master of Medical Education (MME) or a university degree in education (Diplom degree in Pedagogy).

If a trainer wishes to teach a course they have not yet taught, then they must audit this course at least once as an observer to become familiar with the course content and teaching.

Since formal qualification does not automatically mean that a person is able to teach competently, there are several questions in the course evaluations that directly address the individual trainer, so that action can be taken if needed. So far there has been no need for this (see below).

#### 2.4. Trainees

**Figure 1:**
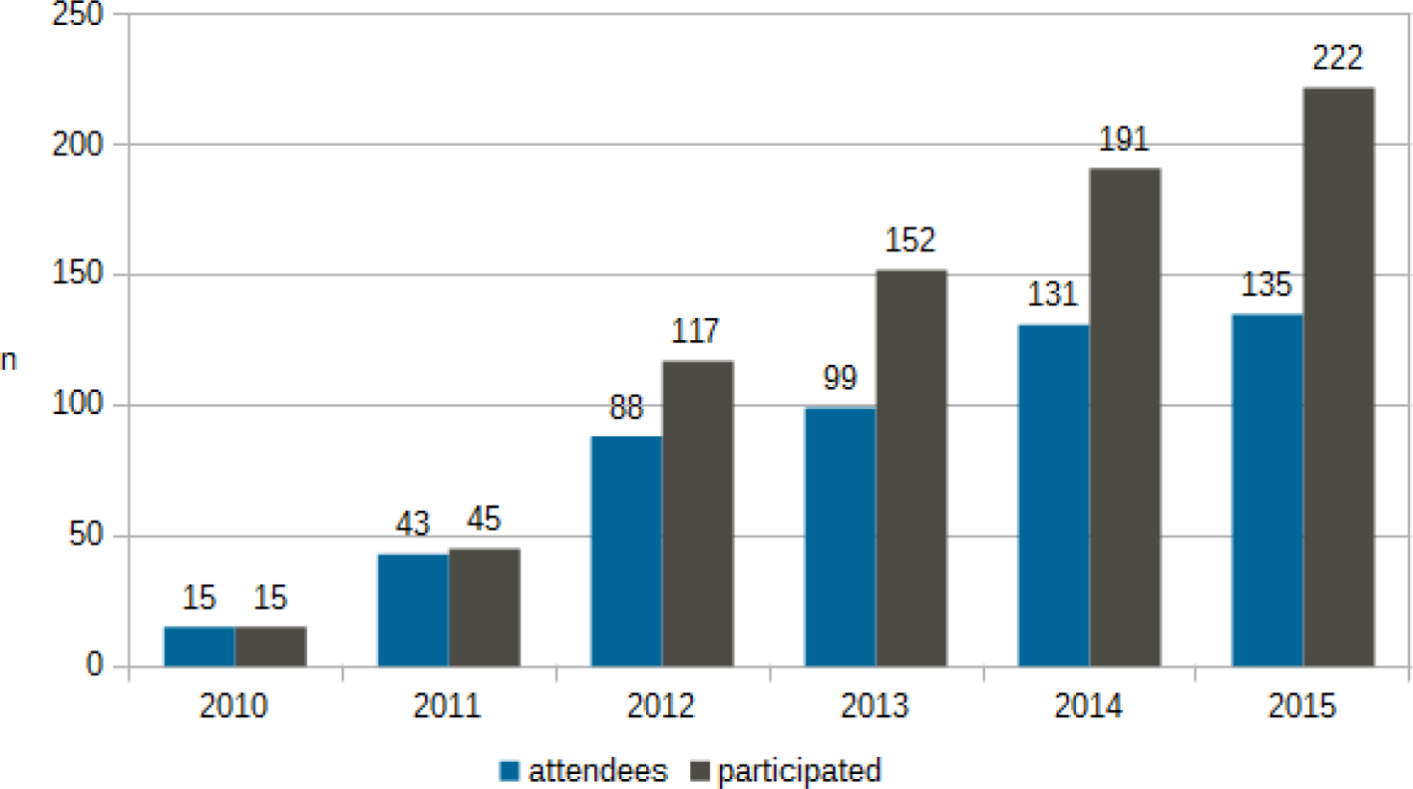
Positive change in participant numbers

A total of 54 people have received certification. Figure 1 (Fig. 1) provides an overview of the growth in programme participant numbers since 2010. The continual increase is clearly reflected in the step-wise increase in the number of necessary courses for registration as university lecturer.

#### 2.5. Evaluation results

The previously mentioned evaluation is a fixed component of the courses. They are administered directly following the end of the course using a questionnaire developed by FAM to meet its particular needs. The questionnaire covers several topics. First, there is a general evaluation of the course with questions about organization and teaching, the subjective assessment of knowledge gain, and the materials used. Second, questions are asked about the course content; and third, the individual trainers are rated. In addition, there are open-ended questions about what the trainees particularly liked or what they found could be improved. All responses are given using a six-point scale spanning a defined range (e.g. “fully disagree” to “fully agree“).

A glance at the overall ratings given for the courses (M=1.6; SD =0.7) and for the trainers (M=1.4; SD=0.6) in the form of standardized academic grades reveals a high degree of satisfaction with the courses that have been taught up to now.

In addition to enabling the highest possible knowledge gain, the teaching content should also have a high degree of practical relevance for the trainees, so that what has been learned may be applied easily to teaching. An important indicator for evaluating the quality of a programme is still the question about whether or not the participant would recommend the programme to others. These three aspects – subjective knowledge gain, practical relevance and recommendation – were selected as examples from all the items contained in the evaluation questionnaire and are presented in table 2 (Tab. 2). For all three items, 85% or more of the total responses for each item rated it with one of the two highest possible evaluative ratings; the lowest level of satisfaction was expressed by only an extremely small minority.

Likewise, the other general evaluations have been positive to date. In contrast, the evaluations of the course contents, the feedback, and the experience of the lecturers have been repeatedly used to switch out or improve individual components. As a result, the original design of individual courses is constantly being revised and optimized. In the basic course, for instance, the material on giving PowerPoint presentations was cut since the participants were already well prepared. In place of this, current developments concerning the National Competency-based Catalogue of Learning Objectives in Undergraduate Medical Education and Dental Education (NKLM and NKLZ) have been included as course topics.

In addition to the subjective assessments, in some cases a content-based exam is given directly before and after the course. To date, this has always revealed a significant knowledge gain. It has even been shown for the basic course that the knowledge was still present after six months [4]. Furthermore, it was possible to determine that the activating methods taught in the courses were used by the trainees in their own classes [5].

## 3. Discussion

Much effort had to be invested before this overall concept could be put into place. The following factors were critical to successful implementation:

- Formal delegation of the task to develop a programme;
- Persuasive and well-founded ideas;
- Existing programmes in other German states (primarily Baden-Württemberg and North Rhine-Westphalia) with matching requirements;
- Support of the Deans of Studies;
- Setting down rules regarding the attendance of medical education courses in the university rules regulations governing lecturer qualification;
- A sufficient number of faculty members interested in teaching and education who, usually during their free time, created the courses and attended numerous meetings;
- The perseverance and dedication of all those involved in the process.

Very strong results have been achieved in terms of satisfaction, self-assessment and the benefits perceived by trainees to their own teaching and knowledge. This is all the more encouraging since most trainees do not attend the courses voluntarily, but rather due to lectureship requirements or, in the case of newly hired faculty members, due to the decision issued by the Faculty Council.

The post-assignments for many courses involve the application of course content to the teaching practices of the trainees. The available documentation and reflective statements very clearly show which individual elements of the courses are put to use and that these are also positively rated.

## 4. Conclusion

Establishing a comprehensive programme in medical education to qualify university lecturers proved to be a long and arduous process. The positive evaluations seen so far and the knowledge gain evident through the pre- and post-course assignments demonstrate that the efforts have been well worth it. This is also confirmed in informal conversations with trainees who report that as a result of the courses they have begun to reflect critically on their own teaching with attempts to improve it. Whether or not and to what extent long-term changes in teaching medical education do take place will need to be explored in future studies. At present, data is being collected from those who have been certified to indicate if and how self-assessed knowledge of good teaching, the academic self-concept as teacher, and perceived personal teaching efficacy have changed following completion of the 120 credits.

## Acknowledgements

We wish to thank Hille Lieverscheidt who with her expertise and ideas enriched the working group during the first conference retreat. We also wish to extend our gratitude to Professor Nürnberger for his support and assistance, particularly during the initial founding phase. Our thanks also go to Professor Sader for his unstinting support in the continued development of FAM. We also thank all those who helped to create and establish the programme and all those are currently active as working group members and lecturers (see table 3 (Tab. 3)).

## Competing interests

The authors declare that they have no competing interests. 

## Figures and Tables

**Table 1:**
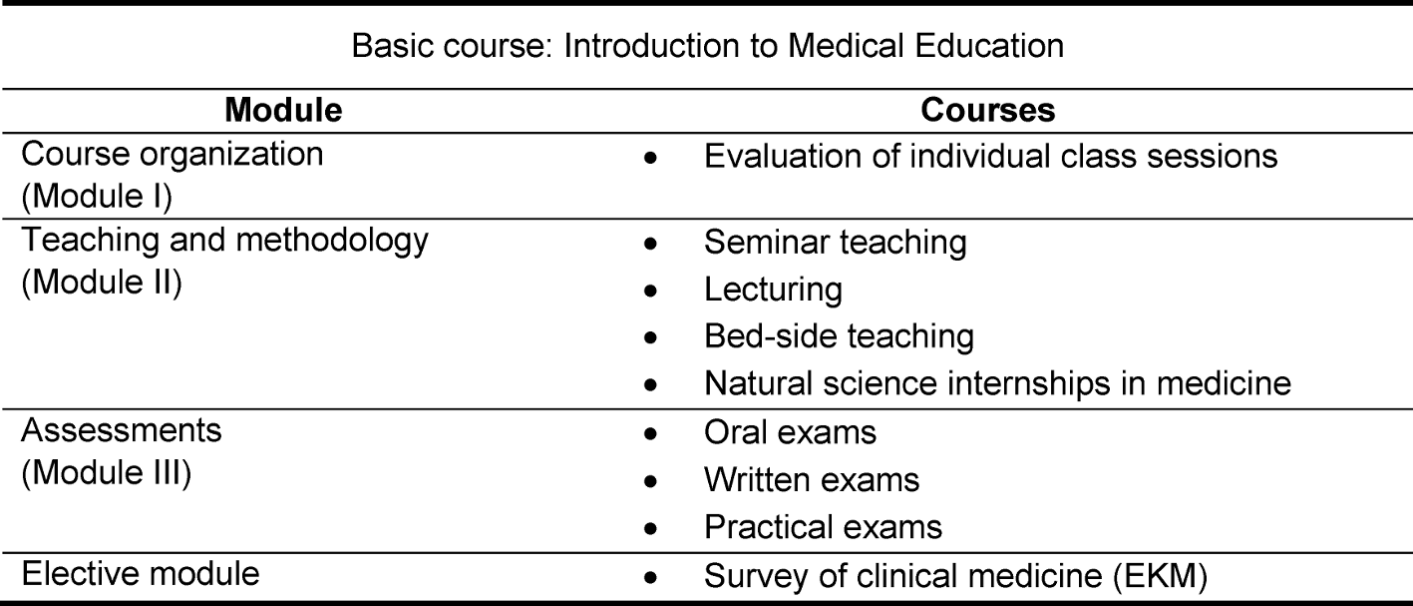
Overview of the modules and the courses offered by FAM

**Table 2:**
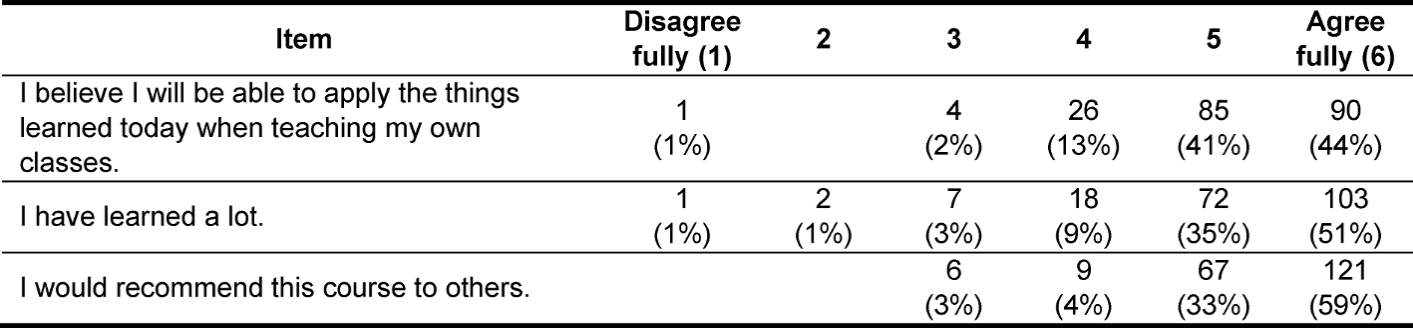
Selected evaluation results for the courses held in 2015 (n=204).

**Table 3:**
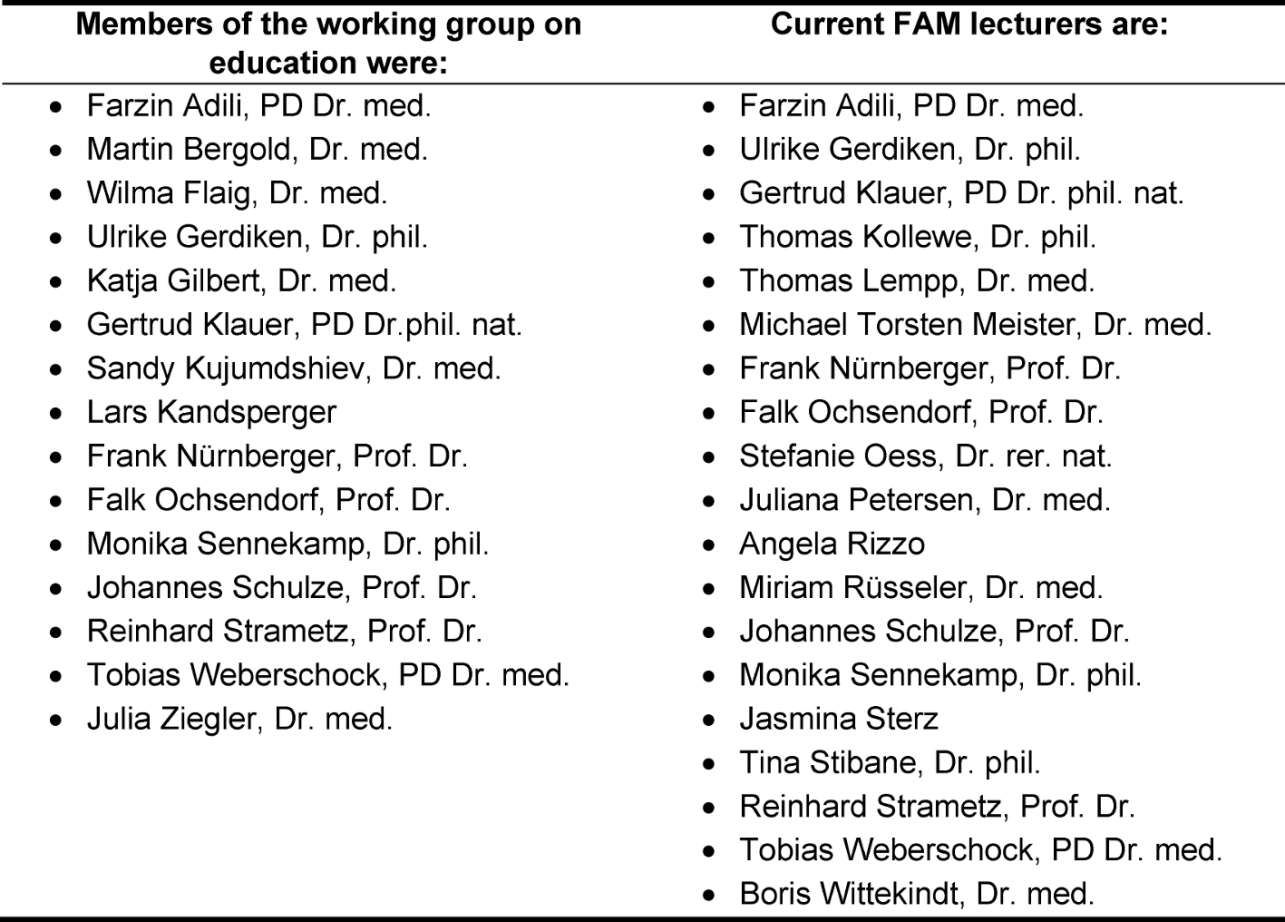
Members of the working group on education and current FAM lecturers

## References

[1] Lammerding-Koeppel M, Ebert T, Goerlitz A, Karsten G, Nounla C, Schmidt S, Stosch C, Dieter P (2016). German MedicalTeachingNetwork (MDN) implementing national standards for teacher training. Med Teach.

[2] Ochsendorf F, Gerdiken U, Sennekamp M (2011). "Die Frankfurter Arbeitsstelle für Medizindidaktik" (FAM) – Von der Idee zur Umsetzung. http://dx.doi.org/10.3205/11gma188.

[3] Lammerding-Koeppel M (2012). Konsenspapier des bundesweiten MedizinDidaktikNetzes zur gegenseitigen Anerkennung von Leistungen.

[4] Ebert T, Sennekamp M, Ochsendorf F (2012). Wie groß ist der Wissenszuwachs nach dem Besuch eines medizindidaktischen Basiskurses und wie nachhaltig ist dieses Wissen?. http://dx.doi.org/10.3205/12gma068.

[5] Kollewe T, Sennekamp M, Ochsendorf F (2015). Einsatz und Bewertung aktivierender Methoden nach dem Besuch des Kurses "Seminardidaktik". http://dx.doi.org/10.3205/15gma248.

